# EANM Dosimetry Committee series on standard operational procedures for internal dosimetry for ^131^I mIBG treatment of neuroendocrine tumours

**DOI:** 10.1186/s40658-020-0282-7

**Published:** 2020-03-06

**Authors:** Jonathan Gear, Carlo Chiesa, Michael Lassmann, Pablo Mínguez Gabiña, Johannes Tran-Gia, Caroline Stokke, Glenn Flux, In collaboration with the EANM Dosimetry Committee

**Affiliations:** 1 Joint Department of Physics, Royal Marsden Hospital & Institute of Cancer Research, Sutton, UK; 2 Nuclear Medicine, Foundation IRCCS Istituto Nazionale Tumori, Milan, Italy; 3 Department of Nuclear Medicine, University of Würzburg, 97080 Würzburg, Germany; 4 Department of Medical Physics and Radiation Protection, Gurutzeta/Cruces University Hospital, Barakaldo, Spain; 5 Department of Diagnostic Physics, Division of Radiology and Nuclear Medicine, Oslo University Hospital, Oslo, Norway

**Keywords:** Dosimetry, Molecular radiotherapy, Iodine-131, mIBG, Neuroendocrine, Neuroblastoma

## Abstract

The purpose of the EANM Dosimetry Committee Series on “Standard Operational Procedures for Dosimetry” (SOP) is to provide advice to scientists and clinicians on how to perform patient-specific absorbed dose assessments. This SOP describes image and data acquisition parameters and dosimetry calculations to determine the absorbed doses delivered to whole-body, tumour and normal organs following a therapeutic administration of ^131^I mIBG for the treatment of neuroblastoma or adult neuroendocrine tumours. Recommendations are based on evidence in recent literature where available and on expert opinion within the community. This SOP is intended to promote standardisation of practice within the community and as such is based on the facilities and expertise that should be available to any centre able to perform specialised treatments with radiopharmaceuticals and patient-specific dosimetry. A clinical example is given to demonstrate the application of the absorbed dose calculations.

## Preamble

The European Association of Nuclear Medicine (EANM) is a professional nonprofit medical association that facilitates communication worldwide among individuals pursuing clinical and research excellence in nuclear medicine. The EANM was founded in 1985.

This guideline is intended to assist practitioners in providing appropriate nuclear medicine care for patients. They are not inflexible rules or requirements of practice and are not intended, nor should they be used, to establish a legal standard of care.

The ultimate judgment regarding the propriety of any specific procedure or course of action must be made by medical professionals taking into account the unique circumstances of each case. Thus, there is no implication that an approach differing from the guidance, standing alone, is below the standard of care. To the contrary, a conscientious practitioner may responsibly adopt a course of action different from that set out in the guideline when, in the reasonable judgment of the practitioner, such course of action is indicated by the condition of the patient, limitations of available resources or advances in knowledge or technology subsequent to publication of the guidelines.

The practice of medicine involves not only the science but also the art of dealing with the prevention, diagnosis, alleviation and treatment of disease. The variety and complexity of human conditions make it impossible to always reach the most appropriate diagnosis or to predict with certainty a particular response to treatment. Therefore, it should be recognised that adherence to this guideline will not ensure an accurate diagnosis or a successful outcome. All that should be expected is that the practitioner will follow a reasonable course of action based on current knowledge, available resources and the needs of the patient to deliver effective and safe medical care. The sole purpose of this guidance document is to assist practitioners in achieving this objective.

## Introduction

The meta-iodobenzylguanidine (mIBG) molecule is a noradrenaline (also called norepinephrine) analogue that selectively targets cells and tumours of the sympathetic nervous system. ^131^I mIBG is indicated to treat inoperable phaeocromocytoma, paraganglioma, carcinoid tumours, metastatic or recurrent medullary thyroid cancer and stage III or IV neuroblastoma [1], and it has been used as a therapeutic agent for over 30 years [2]. For most pathologies, surgery is the first-line therapy, but if radical surgery is not feasible, other options are chemotherapy and external beam radiotherapy, and patients frequently present for ^131^I mIBG therapy if refractory, or following relapse from previous therapy lines.

The challenges for treatment, imaging and dosimetry with ^131^I mIBG are exacerbated by the wide variation in patient status in terms of age and disease burden, which is usually stage III or stage IV. Patients with neuroendocrine cancer can present with tumour volumes ranging in diameter up to 10 cm or more. Larger volumes can exhibit heterogeneous uptake, despite the relatively limited spatial resolution of ^131^I imaging, and it is likely that treatment efficacy is dependent on this distribution. Activity-limiting toxicity is primarily governed by the absorbed doses delivered to the red marrow and, for repeated administrations, to the liver [3]. For neuroblastoma, absorbed doses delivered to the whole body have been used as a surrogate measure of red marrow absorbed dose and have been shown to correlate with toxicity [4, 5]. Although ^131^I mIBG has conventionally been administered based on fixed or weight-based activities, treatments are now increasingly tailored to individual patients based on bio-kinetics, particularly the whole-body absorbed dose [4, 6, 7]. Results from a recent European survey of 208 centres indicated that 59% of centres providing ^131^I mIBG therapies for neuroblastoma perform some level of dosimetry with 18% utilizing dosimetry-based activity prescriptions [8].

In dosimetric studies that have been conducted, a wide range of whole-body and lesion absorbed doses have been observed from fixed or weight-based administrations of activity [9], and methods for performing dosimetry have differed. A variable response to treatment has been reported and although the treatment is usually given with palliative intent, complete responses have occasionally been reported [10, 11]. A wide range of treatment protocols are followed, using either fixed activities (ranging from 3.7 GBq to 11.2 GBq), weight-based administrations considering a maximum level of 444 MBq/kg in the absence of stem cell rescue, to be modulated according to the haematological reserve, and 666 MBq/kg if stem cells were stored [12], or according to whole-body absorbed doses [4, 7]. An extremely wide range of absorbed doses have been quoted in the literature to tumours and to normal organs [9, 11]. Treatment planning based on pre-therapy dosimetry calculations with I-124 and I-123 have been proposed [4, 13–15]. However, I-123 is prone to potential uncertainties due to the short physical half-life of the isotope compared to ^131^I [16] and I-124 mIBG which are not readily available.

Compared to other molecular radiotherapy treatments, relatively few patients are seen at any one centre. Multi-centre trials are therefore necessary to investigate existing and novel treatment options. For this reason, it is essential that dosimetry methods employed are standardised, verifiable and reproducible. Patients receiving ^131^I mIBG therapy are usually kept in isolation for a number of days, consistent with national legislation, which afford the opportunity to acquire a number of measurements and scans without undue inconvenience to the patient. There is strong evidence to suggest that treatment response is correlated with the absorbed doses delivered rather than with the administered activity [4, 5, 17]. The aim of this guideline is to provide advice for the calculation of absorbed doses delivered to the whole body, liver and radiologically and scintigraphically measurable lesions following therapy. Whole-body dosimetry is recommended as a provisional tool for subsequent treatment planning, while lesion dosimetry can be used as a measure of treatment efficacy, whilst organ dosimetry can be used as a measure of treatment toxicity.

## Whole-body dosimetry

As recommended by the EANM procedure guidelines for ^131^I mIBG therapy, adequate measures need to be implemented to block the retention of free radioiodine in the thyroid [1]. Then the organ that limits the activity to be administered is mainly the red marrow. To perform red marrow dosimetry, blood-based methods can be performed except for those patients with marrow involvement, for which image-based methods are required [18]. However, direct blood withdrawal is difficult in paediatric patients, and on the whole, it implies repeated prolonged contact with patients administered with high activities, which should be avoided from the viewpoint of radiation protection. Thus, as there is evidence to show that whole-body dosimetry correlates with marrow toxicity, whole-body dosimetry may be performed as a surrogate for red marrow dosimetry. A mean value of 1.6 has been found for the ratio between whole-body and red marrow absorbed doses in children treated for neuroblastoma and adults treated for neuroendocrine tumours [19]. A similar approach has been adopted for ^131^I tositumomab (Bexxar) treatment which is administered according to a whole-body absorbed dose of 0.75 Gy [18]. However, whereas treatment planning for Bexxar assumes only 2 decay phases, for ^131^I mIBG treatment, a range of decay phases has been observed [4].

To determine whole-body absorbed dose, the activity remaining within the patient should be measured periodically post administration of the radiopharmaceutical [20]. The most straightforward and reliable method to monitor the activity remaining in a patient is to use a radiation detector positioned at a fixed distance from the patient. Care must be taken to ensure that the distance and position between the patient and the counter are reproduced for each reading. Scintillation camera scans are unreliable due to the difficulty in characterising and correcting for dead-time-related count losses. Furthermore, whole-body scanning on a scintillation camera is considerably more time consuming than collecting probe measurements and only allows for collection of a limited number of time points. The approach recommended is to monitor the count rate from the patient using an energy compensated Geiger–Muller counter connected to a scaler rate meter with count rate integrator placed at 2 m from the patient. A set-up for this approach, using a ceiling-mounted counter that enables the patient to lay down in a reproducible position, has been described by Chittenden et al. [21]. This has the advantage that a large number of data points may be acquired without imposition on a busy nuclear medicine department. For systems designed within the treatment facility, radiation exposure to staff is also reduced as patients do not need to be transported through the hospital. A sodium iodide gamma probe connected to a multi-channel analyser can also be used although the linearity and dead time characteristics of the probe should first be determined to ensure suitability for measurements at high count rates. Dose rate meters can also be used provided the output is sufficiently precise for the expected range observed during the treatment course; a dose rate meter with the functionality to average over a set time frame is advantageous for fluctuating dose rates at low activities. Whichever detection system is used, it should be suitable for the range of activities of interest, typically from 10–30,000 MBq of ^131^I. System dead time should be adequately charecterised and, if necessary, count losses due to dead time corrected for using a similar approach to that described in the dead time section of this guideline.

The steps required to perform accurate whole-body dosimetry using this method are as follows:

### Background readings

A first background rate reading should be acquired prior to administration. Integration of the count rate to determine a sufficient number of counts (> 500) should be obtained to maintain the Poisson noise to less than 5%. In practice, and with a suitable counter, this is possible with readings of no more than 10 min. Where possible during the course of treatment, or if contamination within the room is suspected, further background readings should be obtained, to properly correct each sequential measurement. Sources of variable background are typically from contaminated bed linen, clothing, the bathroom or other iodine administrations in nearby rooms without sufficient shielding. Patient urine left in the nearby WC should be washed, and plastic bags in the case of catheterized patients and soiled nappies should be removed from the room or an adjacent bathroom before measurements are acquired.

### Activity measurement

Measurements should be made of the administration vials or syringe and residues in any administration devices, tubes and syringes accounted for. The net administered activity should be determined in an activity meter calibrated for ^131^I to national standards.

### First measurement

The first patient reading should be acquired immediately following administration and before the first void to obtain the baseline to which further measurements will be normalised. If a previous treatment has been performed (in case of repeated therapies, a patient measurement should be performed before administration so that the activity remaining from the first therapy can be accounted for. Where feasible, the geometric mean of the count rate from anterior-posterior and posterior-anterior positions should be acquired, although this is not sufficiently essential to justify undue inconvenience to a patient who may be attached to external lines. It is recommended to acquire multiple measurements at each time point to obtain an average reading. As with background readings, a sufficient number of counts (> 500) should be obtained to maintain the Poisson noise to less than 5%, which can usually be achieved within 60 s.

### Patient-counter geometry

Readings from ceiling-mounted counters should be acquired with patients lying in the same position. If possible, the bed should be lowered to a horizontal position, and supporting pillows should be removed. Particular care should be taken with paediatric patients to ensure that they are in the same position on the bed for each measurement and the bed should not be moved with respect to the counter between readings. In the case of using a hand-held counter, it is essential to ensure that the patient-counter geometry is reproduced as accurately as possible, as the inverse square law effect can significantly impair the accuracy of calculations. A distance larger than 2 m reduces the relative error derived from mispositioning. For example, a 10-cm lateral positioning error can cause a 1% reduction in intensity measured at a counter 1 m from the patient, yet the error for a detector placed at 2 m would only be 0.2%. The relative error from a change in distance between patient and detector is much larger with a 10-cm misspositioning giving rise to a 23% relative error at 1 m and 11% at 2 m. These errors could possibly increase depending on the type of collimation used around the detector. Shielded or collimated detectors will reduce possible cross talk from activity outside of the patient. However, they will also be more prone to positional differences. Patient positioning using a spacer, against a wall or with feet positions drawn on the floor can help improve reproducibility.

### Second measurement

The second reading should be acquired immediately after the first void to determine the level of administered activity that is immediately excreted.

### Subsequent readings

Readings should ideally be taken as often as possible, ideally every 2 h for the first 24 h whilst excretion is most rapid, and then every 4–6 h thereafter, subject to the patient being awake and in accordance with reproducible positioning. This will generally require the omission of readings taken at night. Patients must void before each reading. A minimum of 3 measurements should be acquired in the first day to adequately characterise the fast excretion phase and at least 2 counts in the following days (early morning and late evening) to produce accurate clearance curves.

### Time–activity curve (TAC)

Decay phases may be determined from the time–activity curve. These may be user-defined or based on statistical tests such as the F-test, Akaike information criterion [22], spectral analysis [23] or curve stripping [24]. Criteria for curve fitting apply to mIBG data as for all time–activity curves used for internal dosimetry, whereby the accuracy of the fit is dependent on the number of the data points, the number of decay phases chosen and the accuracy of each data point acquired. Where possible, a minimum of 3 data points should be used to define each potential phase, and the timing of each measurement should be chosen to adequately characterise the exponential (usually < 0.5, 1 and > 3 half-lives). This is easily achievable using the external counting system described. Integration of the curve from *t* = 0 to ∞ is used to determine the time-integrated activity, $\overset{\sim }{A}$.

### S value

The relevant *S* value may be determined from the patient mass. An empirical equation for patient-specific whole-body *S* values, based on patient weight, can be generated by interpolating data from the newborn, 1-year-old, 5-year-old and adult phantoms [25, 26], yielding the formula

1 $$ S\left({r}_{\mathrm{WB}}\leftarrow {r}_{\mathrm{WB}}\right)=1.34.\times {10}^{-4}{m}^{-0.921}\kern1em \left(\mathrm{Gy}{\mathrm{MBq}}^{-1}{\mathrm{h}}^{-1}\right), $$

where the *S* value, *S*(*r*_WB_ ← *r*_WB_) is the radionuclide-specific quantity representing the mean absorbed dose rate to the whole body after administration per unit activity present in the patient assumed to be uniformly distributed in the body, and *m* is the patient’s mass in kilograms.

### Absorbed dose calculations

The mean absorbed dose, *D*(*r*_WB_, *T*_*D*_) to the whole body over a defined dose-integration period *T*_*D*_ may then be calculated using the MIRD equation

2 $$ D\left({r}_{\mathrm{WB}},{T}_D\right)=\overset{\sim }{A}\left({r}_{\mathrm{WB}},{T}_D\right)S\left({r}_{\mathrm{WB}}\leftarrow {r}_{\mathrm{WB}}\right), $$

where $\overset{\sim }{A}\left({r}_{\mathrm{WB}},{T}_D\right)$ is the time-integrated activity (or total number of nuclear transformations) in the patient over a dose-integration period *T*_*D*_. For a repeated administration schedule, the planned administered activity, *A*_*i*_, for administration *i* to deliver a target whole-body absorbed dose, can be planned from the whole-body absorbed dose of the first administration according to

3 $$ {A}_i=\frac{A_1}{D\left({r}_{\mathrm{WB}},{T}_1\right)}\frac{\left(D\left({r}_{\mathrm{WB}},{T}_D\right)-D\left({r}_{\mathrm{WB}},{T}_1\right)\right)}{\left(N-1\right)}, $$

where *D*(*r*_WB_, *T*_1_)and *A*_1_ are the whole-body absorbed dose and the administered activity from the first treatment fraction, respectively, and *N* is the planned number of treatment administrations.

## Tumour and normal organ dosimetry

Absorbed dose calculations for tumours and for normal organs-at-risk follow essentially the same protocol. Multiple whole-body scans have frequently been used for patient dosimetry, and these have the advantage that the full distribution of activity within the body can be visualised. However, to perform the most accurate dosimetry possible for ^131^I mIBG therapy, SPECT or, ideally, SPECT/CT imaging is highly recommended due to the variable localisation of tumours and organs which can entail substantial contributions from active uptake in under and overlying tissues. This is particularly true for larger lesions which can also demonstrate heterogeneous uptake. As gamma cameras are not optimised for high-activity imaging of high-energy gamma emitters, several procedures must be performed to determine the activity imaged in each scan prior to performing dosimetry calculations.

### Quantification/calibration

Image quantification, particularly for ^131^I, has been the subject of significant research for many years. Nevertheless, it has been demonstrated that ^131^I imaging data may be quantified with sufficient accuracy to produce clinically meaningful results [5, 25]. A number of variations have been employed to calibrate the count data acquired [25, 27–31]. One approach is to obtain sensitivity measurements from a point source in air and use this sensitivity factor in the reconstruction to obtain an image in units of activity concentration. However, the sensitivity measurement is sensitive to septal pentetration which varies with source to collimator distance. The reconstruction accuracy is also highly dependent on corrections for scatter, attenuation, system spatial resolution and collimator detector response. For this reason, it is recommended to calibrate post-reconstruction with a volumetric phantom (such as a 20-cm-diameter Jaszczak cylinder) filled with a known concentration of activity [32]. The advantage of this approach is that it accounts for variations in local protocols for image acquisition and reconstruction software. For the calibration protocol, it is essential that the ^131^I activity, concentration and phantom volume are accurately known and, therefore, stock volumes should be measured in an activity meter calibrated to national standards. Activity within the phantom should be sufficiently low to ensure that there is no relevant dead-time-related count losses during scanning. It is essential to image and process the calibration phantom data in the same way as the patient data (employing the same reconstruction parameters including scatter and attenuation correction) [31].

The camera calibration factor, *Q*, is determined from the count rate within a volume of interest (VOI), 75% the physical phantom size, centred within the reconstructed phantom image volume, divided by the exact activity concentration at the time of scanning and the VOI volume;

4 $$ Q=\frac{{\dot{C}}_{\mathrm{VOI}}}{A_{\mathrm{VOI}}}, $$

where ${\dot{C}}_{\mathrm{VOI}}$ is the count rate measured in the VOI, and *A*_VOI_ is the decay-corrected activity in the VOI, i.e. the activity concentration multiplied by VOI volume. The size and position of the VOI should be chosen such that the observed count rate is not affected by partial volume effects or statistical noise. This can be tested by using multiple small VOIs within the larger VOI volume and ensuring that a similar value for *Q* is obtained in all cases.

### Partial volume correction

In addition to the sensitivity measurement, it is also recommended to characterise partial volume effects by imaging a series of phantoms with different volumes and levels of activity that mimic the range for which dosimetry may be carried out. This phantom series should comprise a set of fillable spheres or cylinders of known volume (ideally up to 60 mm in diameter for ^131^I-based imaging which is typically performed with high energy collimation), placed within the larger phantom. Care should be taken to ensure minimal cross talk of counts between inserts, which may require larger inserts to be scanned separately. A recovery term for each insert volume, which can be used to volume-dependently correct for partial volume-based count losses, is given by

5 $$ {R}_i=\frac{{\dot{C}}_i}{QA_i} $$

where ${\dot{C}}_i$ is the observed count rate measured within a VOI matching the true volume of the insert *i* and *A*_*i*_ is the known activity within insert *i*. When subsequently applying the recovery factor to clinical data, any difference between the shapes of the VOI to that of the recovery phantom will influence the accuracy of this correction, as will differing target-to-background concentrations. In some instances, it may be more appropriate to use anthropomorphic-shaped inserts specific to the patient.

### Dead time

The high count rate encountered from patients treated with ^131^I can cause camera dead-time-related count losses. This is particularly pertinent when imaging during the first days post administration, whereby the counts acquired by the camera do not increase linearly with increasing activity [31, 33, 34]. The behaviour of cameras can vary widely so it is essential to characterise the camera used for therapy imaging. A dual source method has been proposed [35], whereby the count rate from a known low activity source is measured with and without the presence of a high activity source. This approach is quick; however, it does not account for the contribution of scatter events that can affect system dead time. An alternative approach entails placing a known source within the field of view and normalising the total counts acquired within the image to those obtained from the source without the patient present. However, this is unwieldy and subject to errors from interference of counts emanating from the source and the patient. It is therefore recommended to characterise the camera for dead time by imaging a source in a scatter medium (such as a water-filled Jaszczak phantom) at varying activity levels [32]. This can be achieved from static acquisitions of a decaying source, although this requires many measurements over a period of weeks. A more practical solution is to repeatedly image a source to which small quantities of known activity are continually (and carefully) added. A detailed description of this methodology and comparative characteristics of different manufacturers is given by [31]. For a paralysable system, the observed count rate measured by a gamma camera, ${\dot{C}}_{\mathrm{obs}}$, is related to the incident count rate, ${\dot{C}}_{\mathrm{inc}}$, and the system dead time, *τ*, as

6 $$ {\dot{C}}_{\mathrm{obs}}={\dot{C}}_{\mathrm{inc}}{e}^{-{\dot{C}}_{\mathrm{inc}}\tau } $$

**Fig. 1:**
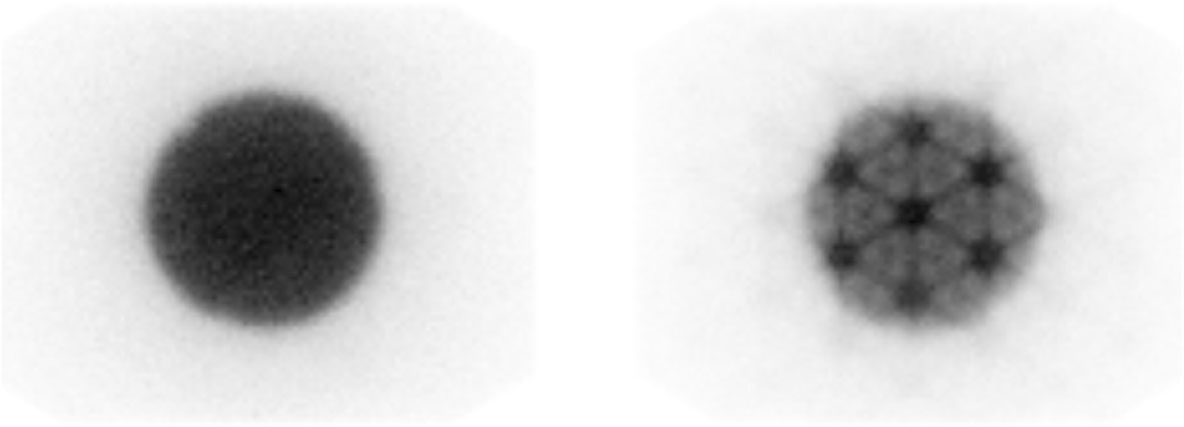
Example images demonstrating typical artefacts that can appear during high count rate imaging

The methodology employed to measure dead time from a decaying source is similar to that described by the National Electrical Manufacturers Association (NEMA) [36] to determine count rate performance. Dead time characterisation obtained from increasing source activities are determined by linearly extrapolating the measured count rates at low activities to that at higher activities. A nonlinear least squares minimisation algorithm can then be used to determine an estimate of *τ* for all values of ${\dot{C}}_{\mathrm{obs}}$ and ${\dot{C}}_{\mathrm{inc}}$. In addition to the measurement of *τ*, it is necessary to determine potential changes in image uniformity that can arise from high count rates. An illustrative example of this is given in Fig. 1 for a uniform cylindrical phantom imaged at low and high count rates. In this example, although an absolute correction for count losses can be made, the tube artefact effect on the high count image renders the image unsuitable for quantitative imaging.

In patient acquisitions, correction for dead-time-related count losses can be applied to each projection, based on the count rate incident at each projection angle. Alternatively, an average correction can be applied (pre or post reconstruction) based on the mean count rate incident over all projections. Correction of the dead-time-related count losses in each pixel, voxel or VOI count rate should then be multiplied by a dead time correction factor, *DTF*, which can be determined iteratively [37] using the measured system dead time and the observed count rate in the patient projection data, ${\dot{C}}_p$.

7 $$ {\mathrm{DTF}}^{i+1}={e}^{\left({\mathrm{DTF}}^i\times \tau \times {\dot{C}}_p\right)} $$

### Data acquisition

Although the design and performance of gamma cameras has changed little in the last 25 years, there are nevertheless differences between systems that can affect results. For example, thicker crystal thicknesses are available for some systems (typically 15.9 mm rather than 9.5 mm) which may render the system more suitable to pre-therapy tracer studies and later-time point therapy imaging but oversensitive to early time point high activities. This may impact the quantitative accuracy of scans acquired shortly after a therapeutic administration. In general, it is recommended that patients are imaged on the same scanner for the entirety of the study. Due to septal penetration of the high-energy photons (364 keV, 637 keV and 723 keV) emitted by ^131^I, a high-energy general purpose parallel hole or ultra-high-energy collimator should be used [38]. Energy windows should be configured to enable triple energy window (TEW) scatter correction to be performed, consisting of a 15–20% window around the main energy peak and 6% windows placed on either side and immediately adjacent to the main window. Wider scatter window widths are not recommended as they may increasingly include scatter events spatially inconsistent with that inside the photopeak, and for the case of the lower window partially cover the 284 keV gamma emission. The camera radial position should be set with auto contouring to ensure optimal spatial resolution.

Data should be acquired into a 128 × 128 matrix or higher with a minimum of 60 projections (or 6° angular steps). The acquisition time for each projection will be dependent on the count rate and on patient comfort. Typical scan times may be as little as 5 s per projection for an initial scan or up to 60 s or more for later scans. The timing of the initial scan will be dependent on the camera dead time characteristics, but should be acquired as soon as possible after administration. Ideally, further scans should be acquired at least daily until either the count rate is too low or until the patient has been discharged and is unable to attend the department for further scans. In practice, a minimum of 3 scans should be acquired to ensure a basic minimum accuracy for subsequent fitting to the time–activity curve. A useful measure to test that the TAC is adequately charecterised is to calculate the time-integrated activity over the time period between the first and last measurements. It is recommended that the fraction of the time-integrated activity between measurements is greater than 80% of the total time-integrated activity when extrapolated from zero to infinity [20].

### Scatter correction

The raw image data should be corrected for scatter using the data acquired in the three energy windows according to the method of Ogawa et al. [39] whereby the scatter-corrected counts (*C*_SC_) in the main window are given by

8 $$ {C}_{\mathrm{SC}}={C}_{\mathrm{peak}}-\frac{W_{\mathrm{peak}}}{2}\left(\frac{C_1}{W_1}+\frac{C_2}{W_2}\right), $$

where *C*_peak_ is the projection counts acquired within in the main window of width *W*_peak_ and *C*_1_/*C*_2_ are the projection counts acquired within the lower/upper energy scatter window of width *W*_1_/*W*_2_, respectively.

In modern reconstruction software, scatter correction is typically automatic and included in the iterative loop. Older software may, however, require subtraction of projections. In addition, some newer software use a simulated scatter image, negating the need to acquire separate scatter windows.

### Attenuation correction

As many neuroendocrine tumours are deep-seated, attenuation correction should be performed according to local protocols. As hybrid SPECT/CT scanners become more widespread, the CT scan can be used for generating an attenuation map, although the lack of CT data should not inhibit the application of dosimetry. If no CT-based attenuation is possible, a relatively simple yet effective method of attenuation correction may be applied according to the method of Chang et al. [40]. For paediatric patients, the abdomen can be considered similar to an adult head and, therefore, the use of a linear attenuation assumption can be justified when compared to common practice for SPECT brain imaging. The attenuation coefficient of 364 keV photons in water is 0.11 cm^-1^ [32], and a reasonable estimate of the body outline can be visibly obtained from the lower energy window scatter image.

### Image reconstruction

Iterative reconstruction methods are recommended. However, it should be noted that results can vary highly depending on the reconstruction parameters used, which should always be clearly reported [41]. Optimal reconstruction parameters for diagnostic imaging do not necessarily provide the most accurate quantification. For this reason, an optimisation of the reconstruction is required. Estimates of the optimal reconstruction parameters can be obtained by examining the calibration acquisition data. Starting from the default clinical settings, the number of OSEM updates (product of iterations and subsets) should be increased and the recovery coefficient, *R*, series should be determined as a function of updates. Ideal reconstruction settings are those when *R* is close to the maximum achievable without significant image noise. The noise level can be studied with a VOI in the large cylindrical phantom, plotted as a function of update number. It may be necessary to adjust the number of updates once clinical data are acquired to ensure image convergence and appropriate noise levels. Many modern reconstruction algorithms offer collimator-detector response corrections which can lead to higher count recovery during reconstruction and suppressed image noise. However, care should be taken to avoid Gibbs ringing artefacts that can manifest with this correction and ultimately misrepresent the distribution of activity [42].

### Volume definition

Absorbed doses may be calculated for either the entire volume of the tumour or normal organ or for any sub-organ volume. Conventionally, mean absorbed doses are calculated over the entire volume of interest. For this approach, results are highly dependent on delineation of the volume. For visibly uniform uptake and where ‘anatomical’ imaging is available (i.e. CT or MRI) with sufficient contrast to delineate the organ or lesion, this may be used. In cases with heterogeneous uptake or insufficient anatomical contrast, ‘functional’ (based on SPECT/PET data) volume delineation may be necessary. A widely used approach is to use an adaptive threshold approach on the SPECT data using factors determined from the calibration data [43, 44].

Once the appropriate VOI is defined, the count rate at each time point ${\dot{C}}_v(t)$ is converted to activity using the appropriate calibration, recovery and (if being applied, post reconstruction) dead time factor

9 $$ {A}_v(t)=\frac{{\dot{C}}_v(t)}{Q\bullet R(v)}.\mathrm{DTF} $$

Voxel-based dosimetry has been proposed as a method for which calculations may be subsequently determined for any defined volume. Many neuroendocrine tumours are of a substantial volume and contain a heterogeneous distribution of activity, visible even within the limitations of ^131^I imaging. Sub-volumes of a region of interest may therefore also be defined. Of potential interest are the maximum and minimum absorbed doses. A relatively simple approach (but also prone to noise-related artefacts) is to identify a single voxel in the sequential scan set that represents the maximum or minimum uptake. An extension of this approach is to identify a sub-volume, consisting for example of a block of 27 voxels that represent volumes of maximum or minimum uptake. This larger volume is potentially less prone to noise-related artefacts compared to reporting a single voxel absorbed dose.

### Time–activity curve (TAC)

Effective half-lives can vary widely from patient to patient and should not be assumed based on a population average. A series of sequential scans is therefore essential. As SPECT has a limited field of view, it may be necessary to obtain two sets of scans if dosimetry is required for widely separated volumes. The time–activity data for the chosen region should be plotted and the area under the curve should be integrated. The method of integration and the assumptions made regarding uptake or decay before the first time point or following the last time point can have a significant impact on the time-integrated activity calculations and should be addressed carefully. It is recommended that extrapolation of the later time points is performed to define the effective decay phase, rather than assuming physical decay.

### Absorbed dose calculation

MIRD *S* values [45] or RADAR dose factors [26, 46], used to convert the time-integrated activity to the absorbed dose, are available for standardised organ geometries but are not defined for tumours, nor for sub-volumes within tumours or normal organs. For the calculation of absorbed doses to normal organs, such as the liver, the adjustment of mass according to patient-specific measurements can improve the accuracy significantly and is recommended [47]. For non-standard organs (such as lesions) the *S* value chosen for the absorbed dose calculation will be dependent on the region of interest chosen. In many cases, a spherical *S* value will be sufficient to provide a reasonable estimate of the absorbed dose. *S* values for individual voxels (dose point kernels) of varying size are available from a number of sources [48] for several radionuclides. It is possible to perform dose calculation beyond the mean absorbed dose, that take into account dose rate and heterogeneity in tracer uptake, such as the biological effective dose (BED) and equivalent uniform dose (EUD). Methods for calculating these parameters is beyond the scope of this guidance document but is well described within the literature [49].

## Discussion

^131^I mIBG treatment, imaging and dosimetry present particular challenges. Patients with neuroblastoma can be as young as 2 years old, whilst adult neuroendocrine patients can be elderly. At present, there are no guidelines to govern the levels of activity to be administered or the frequency of administrations, and treatment regimens are known to vary widely [9]. There is also dispute concerning the potential benefit of concomitant administration of chemo-radiosensitisers, and ^131^I mIBG has been administered in conjunction with cisplatin [50], topotecan [7], and irinotecan [51]. It is likely that optimal administration protocols can only be identified in large-scale multi-centre trials.

There is no common pattern of previous treatments or stage of presentation and tumours can vary widely in size and location. Treatment protocols tailored to whole-body absorbed doses, such as the protocol for neuroblastoma in which 444 MBq/kg are administered in a first fraction in order to reach a whole-body absorbed dose of 4 Gy in two fractions [7], can result in administered activities in excess of 30 GBq. This entails challenges for quantitative imaging on technology that was designed for diagnostic levels of Tc-99m and careful radiation protection considerations. Some centres may have legal restrictions in the maximum activity to be administered, which may force them to decrease the total activity to be administered if the treatment is given in two fractions. A recent study [52] analysed from a radiobiological viewpoint the benefits of different schedules with more fractions without decreasing the total activity to be administered. The study demonstrated that although the tumour BED could decrease by up to 12% when delivering 4 Gy whole-body doses over more than two fractions, this decrease was modest compared to reducing the overall administered activity.

At present, the calculation of absorbed doses for any given radiopharmaceutical, and for any given patient, necessitates several assumptions and simplifications. These are unavoidable, but should not prevent absorbed dose calculations from being performed. The methodology followed, and the assumptions made, should be reported with the results of the absorbed dose calculations. Owing to different facilities and logistical considerations at different centres, and to a lack of multicentre trials, there can be no definitive protocol to cover all aspects of imaging and dosimetry at present. It is recommended that results are reported according to the EANM dosimetry reporting guidelines [41], and that uncertainty estimates are made and quoted alongside the dose calculation [16, 53].

Whole-body dosimetry is a simple and effective means of tailoring an administered activity in treatment planning. Due to the simplicity of the measurement process, a large number of data points can be acquired, which inevitably reduces the overall uncertainty in the dose calculation. *S* values can be determined by a simple mass measurement of the patient and also has very small measurement uncertainty. Uncertainty in organ and lesion dosimetry are considerably larger particularly due to the unavoidable delay between administration and the first imaging time point whereby uncertainties in time-integrated activity are dominated by the ability to adequately characterise the time–activity curve. Organ and lesion delineation is also particularly challenging due to the low resolution of ^131^I imaging and small size pf paediatrics. Uncertainties can be reduced by using good-quality high-resolution anatomical imaging to aid delineation. This is demonstrated in the clinical example where uncertainties in absorbed dose calculations are within 15 %.

Dosimetry scans can be particularly burdensome especially for some very young paediatric patients with neuroblastoma. It is therefore possible that some children may require a form of sedation or anaesthesia or distraction techniques to tolerate the treatment and scanning regimen. Careful consideration of the risk–benefit to these patients should therefore be taken when selecting a treatment and dosimetry approach. Chittenden et al. [21] reported that with an optimised environment paediatric patients found whole-body measurements easy to tolerate and a large number of data acquisition points were achieved.

Despite the challenges and uncertainties involved, the value of dosimetry in mIBG therapy cannot be doubted. This was demonstrated as early as 1992 by a study to determine optimal treatments on a patient-specific basis [6]. There is sufficient evidence for a correlation between whole-body dose and toxicity to warrant multi-centre studies. Moreover, some studies have reported on a correlation between the lesion absorbed dose and the response to the ^131^I mIBG therapy in patients treated for neuroblastoma and phaeochromocytoma [5, 54, 55].

Personalised treatment planning will continue to evolve. The relative absorbed doses delivered from consecutive therapies in the same patient [56] can be used to inform whether subsequent treatments continue to deliver a clinically meaningful absorbed dose and whether this should be modified in an adaptive planning approach. A tracer (i.e. a small and only used for pre-therapeutic dosimetry) activity of ^131^I mIBG could also be used to optimise the activity to administer in relation to the absorbed dose to lesions and normal organs. Moreover, despite being regarded as a ‘dirty isotope’ [14], the use of I-124 mIBG in PET imaging will increase for ^131^I treatment planning, as it offers a degree of quantitative accuracy not achievable with ^131^I SPECT imaging [13, 57, 58].

A number of challenges remain that will emerge as dosimetry becomes more commonplace. These include the application of voxel dosimetry, the reporting of which is not standardised. Forthcoming developments hold great promise for this therapy. In addition to radiobiology, these include the potential for radiosensitiers, including cell repair inhibitors [59], carrier-free mIBG, and imaging and dosimetry with I-124 mIBG. There is also a strong potential for combined therapies with external beam radiotherapy as well as chemotherapy [60], immunotherapy [59] and other molecular radiotherapy treatments.

## Conclusions

Imaging and dosimetry for ^131^I mIBG can be of significant clinical benefit. At present, absorbed doses are calculated for only a small number of patients, although, where studies have been performed, a wide range of absorbed doses has been reported. The methods outlined in these guidelines are not prescriptive, but nevertheless aim to harmonise data collection among centres, in order to obtain comparable data. Methods will be improved by refined techniques. However, they should be within the range of all cancer centres that offer targeted molecular radiotherapy.

## Example

In this example, the dosimetry methodology for a paediatric neuroblastoma patient treated with two fractions of ^131^I mIBG is presented. The protocol described aims to deliver a total whole-body (WB) absorbed-dose of 4 Gy, delivered in 2 fractions 2 weeks apart. Lesion and normal organ doses following each fraction are also calculated.

An activity of 6893 MBq was administered in the first fraction based on a patient weight of 17.5 ± 0.5 kg. Whole-body activity retention measurements were performed to calculate the whole-body absorbed radiation dose. The second fraction of mIBG was administered according to the measured absorbed whole-body dose of the first fraction (calculated to 10,566 MBq, see below).

### Whole-body dosimetry

**Fig. 2:**
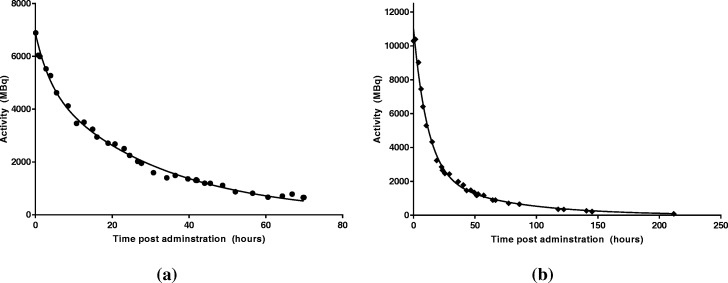
Whole-body retention data from first (**a**) and second (**b**) adminstration

During the first treatment fraction, a fixed geometry Geiger counter above the patient bed enabled the patient’s carers and ward staff to take readings after every void. On average, 8 measurements per day were acquired for 2 × 60 s each in supine patient position (i.e. the patient lying flat on the patient bed). The acquired whole-body retention curve is given in Fig. 2a. An extra sum of squares *F* test indicated a bi-exponential function was most appropriate to fit to the data, *F* (DFn, DFd) = 41.8 (2, 80), *P* value < 0.0001. A patient-specific *S* value was determined using expression (1) and the patient mass to give *S*_WB ← WB_ = 9.60 × 10^−6^ GyMBq^−1^*h*^−1^ . Expressions (2) and (3) were used to calculate a whole-body absorbed dose of 1.58 Gy from the first administration (0.23 Gy/GBq) with a planned second administration of 10,566 MBq to give a total 4 Gy absorbed whole-body dose. Dosimetry factors for the second administration and the associated time–activity curve are given in Table 1 and Fig. 2b, respectively.

**Table 1:** Dosimetric parameters from whole-body data following both administations

	1st Administration	2nd Administration
$\overset{\sim }{A}$ (MBq h^-1^)	164,500 ± 2600	255,093 ± 13,000
*S* value (Gy/MBq.h)	(9.6 ± 0.3) × 10^-6^	(9.60 ± 0.3) × 10^-6^
Absorbed dose (Gy)	1.58 ± 0.03	2.45 ± 0.09

### Camera dead time

**Fig. 3:**
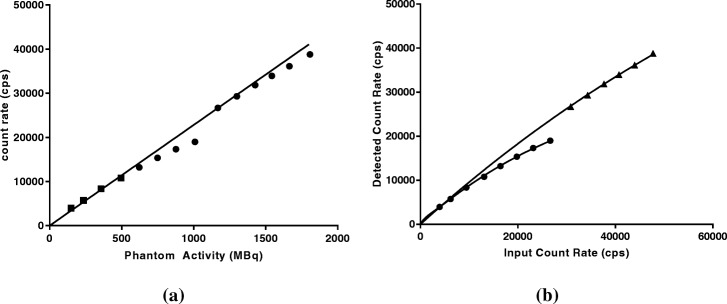
**a** Camera dead time data for increasing activities showing linear fit to low activity data points (solid line) and **b** dead time curve fitted with the appropriate dead time function (solid line)

Prior to patient imaging, system calibration and dead time factors were determined. The Philips FORTE gamma camera (9.5-mm crystal thickness) operates a ‘high-count rate mode’, which activates at count rates above 20 kcps. Therefore, dead time models were required for each phase in the dead time curve. Graphs of detected count rate vs. phantom activity are shown in Fig. 3a. A linear fit to the low-activity data points (< 10,000 cps) was used to estimate values of input count rate for the corresponding phantom activities (Fig. 3b). Values of *τ* of 12.8 and 0.47 μs were then determined by fitting expression (6) to the corresponding operation modes.

### Quantification

System calibration was performed using a cylinder of water 20 cm in diameter and 12 cm in height containing 56.5 MBq of ^131^I. Images of the phantom were acquired and reconstructed using the parameters described below for 40 s per projection. Reconstructed counts with a central VOI and known activity concentration (15 kBq/ml) were used in expression (4) to determine a calibration factor of *Q* = 29.2 cps/MBq .

**Fig. 4:**
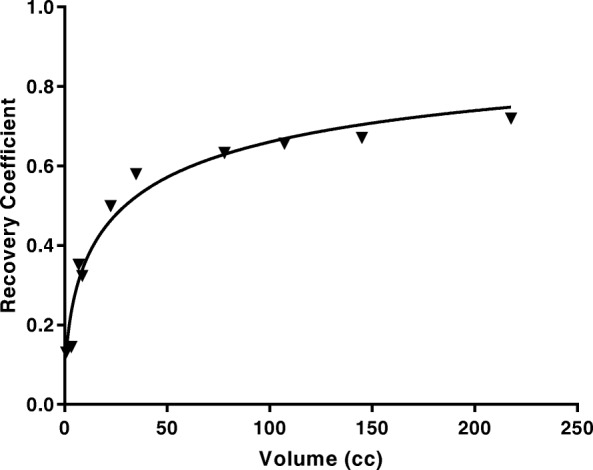
System recovery curve for ^**131**^I derived from different-sized cylindrical inserts

Recovery factors were determined using a set of cylindrical phantoms with volumes ranging from 1 to 220 ml placed within the larger cylinder. The activity concentration within the cylinder inserts was 1.98 MBq/ml at preparation. Each insert was scanned independently and the time per projection was changed between 10 and 80 s to ensure sufficient counts within the projection data. The decay-corrected activity concentration and the reconstructed images were used with expression (5) to generate the recovery curve shown in Fig. 4.

### Lesion and organ dosimetry

**Fig. 5:**
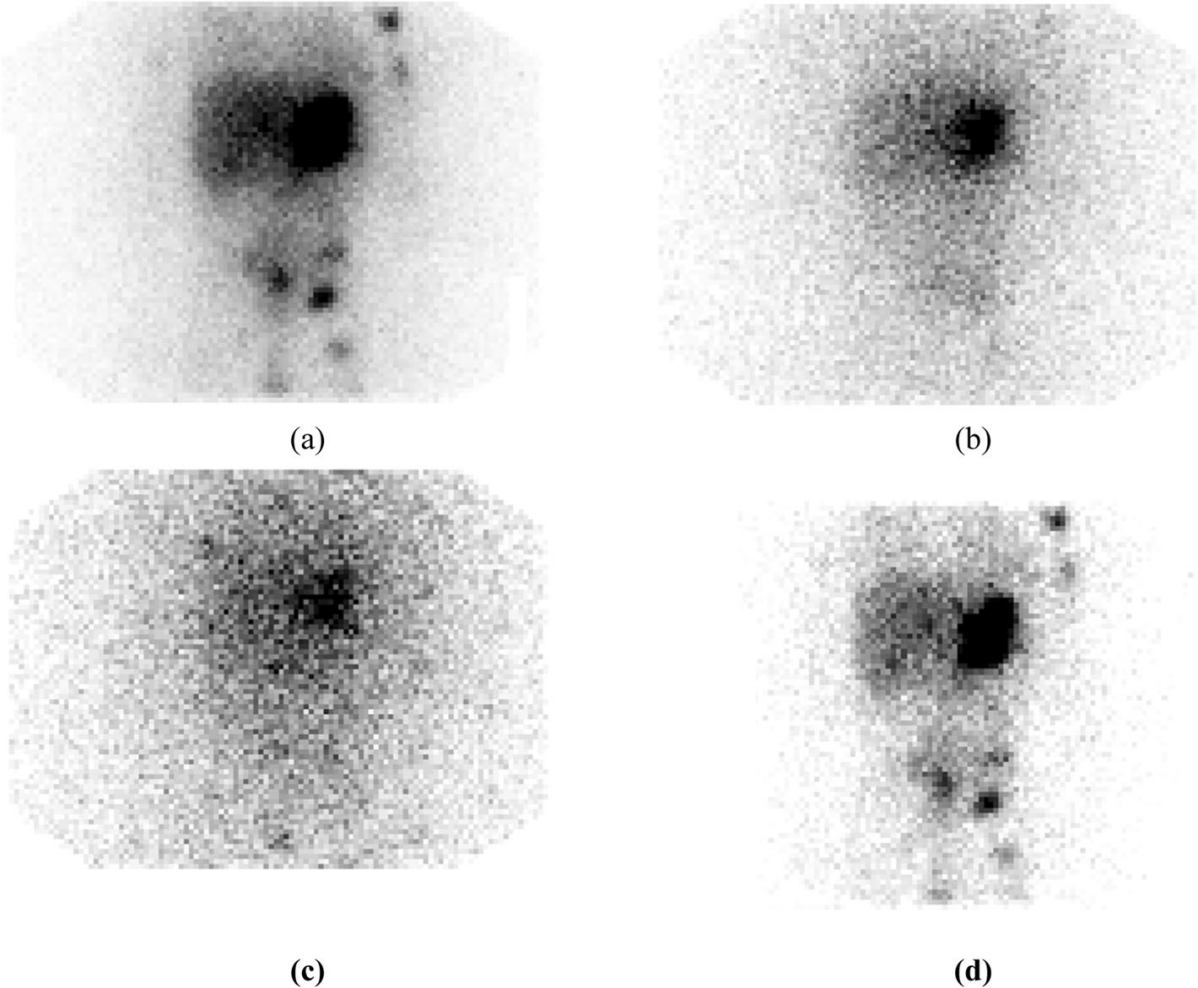
Example projection data aquired in main window (**a**), low-energy scatter window (**b**), high-energy scatter widow (**c**), and after triple-energy window scatter correction (**d**). Each image is scaled to the local maxima and minima on a linear grey scale

Patient SPECT imaging was performed 2, 5 and 7 days following the first ^131^I administration and 2, 5, 6 and 9 days post the second administration on a Philips FORTE gamma camera using the ADAC high-energy-general-purpose (HEGP) collimators. The energy window was selected at the 364 keV photopeak, with a width of 20%. Two scatter windows were also acquired either side of the main window, with a width of 6% each. Sixty-four angular views were acquired for 10 to 40 s according to the count rate. TEW scatter correction was applied. Example projection data (3 energy windows and projection after scatter correction) are given in Fig. 5. The camera-acquired data in step-and-shoot mode following an elliptical orbit around the patient with a 128 × 128 matrix giving a pixel size of 4.67 × 4.67 mm^2^. The average projection count rate within the main window for each dataset was measured and expression (7) was used to determine dead time correction factors, DTF, for each scan. The maximum count rate measured was 25 kcps which resulted in the camera operating in high-count rate mode with a DTF of 1.01 (i.e. 1% dead time losses). All other scans were below the system ‘high-count rate mode’ cutoff, and ranged from 1.6 to 15 kcps. DTFs ranged from 1.02 to 1.29 for these count rates.

Scatter-corrected images were reconstructed iteratively using OSEM with 8 iterations and 16 subsets. A uniform attenuation correction was applied based on the patient size. Following reconstruction, the SPECT datasets were filtered using a Butterworth filter of order 5 cutoff frequency 0.9 cycles/cm and registered to previously acquired CT and MRI scans.

**Fig. 6:**
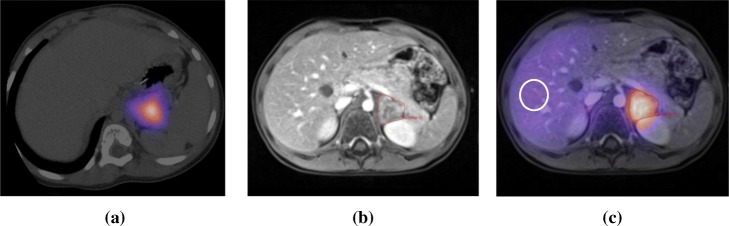
Image data depicting SPECT/CT (**a**), MRI alone (**b**) and fused SPECT/MRI (**c**). The lesion can clearly be seen as the volume with intense uptake adjacent to the left kidney (delineated in **b** and **c**)

The abdominal mass and liver were manually delineated on the anatomical datasets for volume determination. For the lesion, an appropriate threshold was then selected to outline a similar-sized VOI on each SPECT image. Due to the uniform nature of uptake within the (healthy) liver, a small (3-cm diameter) spherical VOI was placed within the liver area to determine the count rate concentration on the respective SPECT datasets. Count rate within each of the VOIs were then converted to activity using the previously acquired calibration factor, *Q*, and the appropriate dead time factor, DTF, for each scan. A recovery coefficient for the appropriate volume was also applied to the lesion VOI. This was not necessary for the liver as the VOI was centrally located where no partial volume effects would occur. Images of the fused datasets are shown in Fig. 6.

**Fig. 7:**
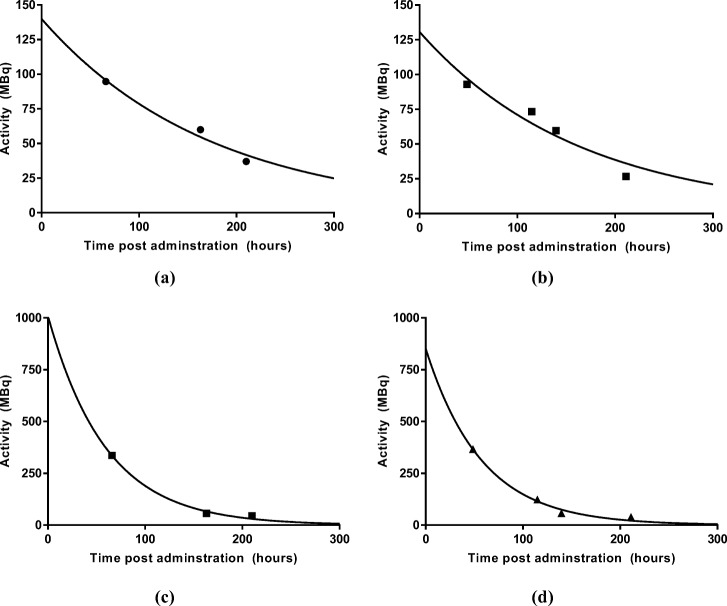
Time–activity curves for the tumour after the first (**a**) and the second administration (**b**). Time–activity curves for the liver after the first (**c**) and the second administration (**d**)

**Fig. 8:**
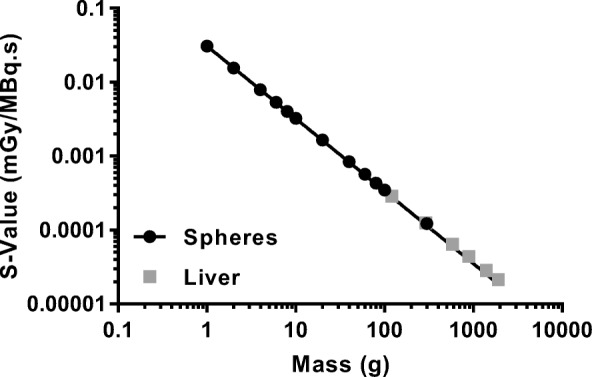
Plot of liver and sphere *S* values used to determine the patient specific *S* value used for dose calculation

Time–activity curves for the lesion and liver after each therapy fraction are given in Fig. 7. A single exponential curve was fitted to the data using an iterative least squares algorithm. *S*-value data for lesion and liver were extrapolated from the unit density sphere models and age-dependent phantoms within the OLINDA/EXM software [46]. These *S* values have been calculated based on the most recent evaluation of electron- and photon-absorbed fractions in spheres of various sizes [61]. A plot of *S* values for the different models is given in Fig. 8 with a fit to the data following the form *S*(*r*_sphere_ ← *r*_sphere_) = 0.031 × *m*^−0.981^ mGy/MBq·s. Absorbed doses for lesion and liver were calculated assuming negligible cross dose between regions. Dosimetry parameters for the liver and lesion for each therapy administration are summarised in Table 2.

**Table 2:** Results of the absorbed dose calculation to normal liver and to tumour

	First administration		Second administration	
	Lesion	Liver	Lesion	Liver
Mass (g)	42.8 ± 2.5	711 ± 16	41.4 ± 2.5	711 ± 16
A_0_ (MBq)	140 ± 23	1010 ± 220	131 ± 30	852 ± 253
Effective half-life (h)	120 ± 22	41.43 ± 10.8	113.7 ± 22.85	39.7 ± 15
Time-integrated activity (MBq·h)	24,280 ± 2389	60,370 ± 5225	21,444 ± 2480	48,853 ± 6741
*S* value (mGy/MBq·s)	(7.8 ± 0.5) × 10 ^-4^	(0.49 ± 0.01) × 10^-4^	(8.0 ± 0.5) × 10^-4^	(0.49 ± 0.01) × 10^-4^
Absorbed dose (Gy)	68.0 ± 7.8	10.6 ± 1.0	62.1 ± 7.8	8.6 ± 1.2

## Liability statement

This guideline summarizes the views of the EANM Dosimetry Committee. It reflects recommendations for which the EANM cannot be held responsible. The recommendations should be taken into context of good practice of nuclear medicine and do not substitute for national and international legal or regulatory provisions.

## Data Availability

The datasets used and/or analysed during the current study are available from the corresponding author on reasonable request.
